# Lactate flux dysfunction in patients with bipolar disorder: preliminary insights from ultra-high field 7T MRSI during task and rest

**DOI:** 10.3389/fnins.2025.1587011

**Published:** 2025-06-04

**Authors:** Michele A. Bertocci, Rasim S. Diler

**Affiliations:** ^1^University of Pittsburgh School of Medicine, Pittsburgh, PA, United States; ^2^Western Psychiatric Hospital, University of Pittsburgh Medical Center (UPMC), Pittsburgh, PA, United States

**Keywords:** lactate, neural metabolism, bipolar disorder, depression, precuneus

## Abstract

**Introduction:**

The symptoms of bipolar disorder (BD) may be characterized as disruptions in energy metabolism, and neural energy availability may serve as a mechanistic marker of BD. Lactate, the end product of glycolysis, is a poorly understood neural energy source that may contribute to the neural dysfunction underlying BD.

**Methods:**

We aimed to assess precuneus lactate availability during an emotion processing task and during rest in a sample of participants with well-characterized, pediatric-onset BD (*n* = 17) and healthy participants (*n* = 8), using 7-Tesla (7T) magnetic resonance spectroscopic imaging (MRSI). The mean age of the participants was 19.2 years (3.8).

**Results:**

In this small sample, we observed that the difference in precuneus lactate availability between the emotion processing task and rest (e.g., lactate flux) was greater in participants with BD [mean = 0.014 (0.041)] than in healthy controls (HCs) [mean = −0.033 (0.028), *t*(17) = 2.64, *p* = 0.017, Cohen’s d = 1.3, Bayes factor_10_ = 3.528]. In addition, we found that this greater difference in lactate availability (task–rest) in participants with BD, particularly those with lower precuneus lactate availability at rest, demonstrated a trend related to elevated depression scores (r = 0.459, *p* = 0.055, Bayes factor_10_ = 1.617).

**Discussion:**

These results suggest, for the first time, using ultra-high-field strength MRSI with a high signal-to-noise ratio, that lactate flux is dysfunctional in well-characterized BD. Our findings highlight the importance of lactate as a mechanistic marker of BD, which may be used to develop novel treatment options.

## Introduction

Bipolar disorder (BD) is a debilitating illness that usually begins in youth, affects 2–5% of the population, and is associated with an increased risk for morbidity, functional impairment, and suicidality (Diler and Birmaher, 2019). An important aspect of the clinical presentation of BD involves energy metabolism-related symptoms, which includes low energy and psychomotor retardation in depression, decreased need for sleep, high energy, and decreased motor hyperactivity in mania. These energy metabolism-related behaviors directly reflect dysfunction in energy processes and have been shown to be more prevalent than mood symptoms in BD (Cheniaux et al., 2014). Indeed, a recent update in the diagnostic criteria for BD in the Diagnostic and Statistical Manual (DSM)-5 included “energy/activity increase” as a required feature of a hypomanic/manic episode, highlighting its importance as an objective clinical marker for an accurate BD diagnosis (APA, 2013). Similarly, abnormalities in brain energetics have been implicated in BD (Kolar et al., 2021), and perturbations of mitochondrial function have been suggested to alter not only brain energy supply and metabolite generation but also energy-related thought processes and behavior changes associated with BD (Buttiker et al., 2022). Findings of mitochondrial dysfunction (Buttiker et al., 2022) and alterations in other metabolites (Scotti-Muzzi et al., 2021) have implicated energy metabolism as a core feature of BD (Chabert et al., 2022); however, these mitochondria-related findings are not specific to BD (Kolar et al., 2021), suggesting that there may be an unidentified key abnormal process in energy mechanisms. One possible energy mechanism involves the underappreciated role of lactate in both the brain and the body (Rabinowitz and Enerback, 2020). The accumulation of lactate in stressed muscles and ischemic tissues has established its reputation as a deleterious waste product, which has limited research interest in its potential key role in brain energetics. However, this reputation may warrant reconsideration in light of *in vitro*, rodent, and human studies (Magistretti and Allaman, 2018).

### A brief history of lactate

Lactate is a naturally occurring energy source produced through the breakdown of glucose in most human cells. Early research characterized lactate as a waste product resulting solely from anaerobic energy metabolism (Fletcher and Hopkins, 1907; Hill et al., 1924; Meyerhof, 1930). Following this characterization, decades of physiological research attributed muscle pain and fatigue to the elevated lactate levels measured after exertion. Studies reporting increased lactate in cardiac patients reinforced this perspective (Wasserman and McIlroy, 1964). In addition, decades of studies have suggested that increased lactate concentrations in the body are associated with poor outcomes after trauma; however, the mechanism of the body’s use of lactate may be misunderstood. Groundbreaking studies in exercise physiology and biochemistry, led by George Brooks’ and Avital Schurr’s independent studies, have demonstrated that lactate is not an *anaerobic* waste product; rather, it is produced under fully aerobic conditions and shuttled to the muscles, heart, and brain as a vital energy source for physical endurance with improved heart and lung functioning (Brooks, 2000; Brooks, 2009; Brooks, 2018; Brooks et al., 1999; Pedersen et al., 2004; Schurr, 2006; Schurr and Payne, 2007) and for neural functioning (Sofroniew and Vinters, 2010; Smith et al., 2003; Descalzi et al., 2019; Glenn et al., 2015; Holloway et al., 2007). This shuttling of lactate throughout the body is referred to as the lactate shuttle theory (Brooks et al., 1999; Brooks, 2000; Brooks, 2002; Brooks, 2009; Brooks, 2018; Brooks, 2020), and in the brain, it is specifically referred to as the astrocyte-neuron lactate shuttle model (Pellerin and Magistretti, 2012; Pellerin et al., 1998; Schurr et al., 1997a; Schurr, 2006; Schurr and Payne, 2007). These models and subsequent rodent and human studies have supported the role of lactate as an important energy source for the muscles, lungs, and brain. Lactate is currently recognized as the endpoint metabolite of glycolysis across all physiological states (Rogatzki et al., 2015; Schurr and Payne, 2007), resulting in the production of adenosine triphosphate (ATP) through multiple mechanisms (Gladden, 2004; Sofroniew and Vinters, 2010; Schurr, 2006; Rouach et al., 2008; Schurr and Payne, 2007; Hu and Wilson, 1997). Lactate is then shuttled for use throughout the body (Bertocci and Lujan, 1999; Schurr, 2006; Schurr and Payne, 2007). Specifically, in the brain, lactate is the preferred neuronal energy source during both high neuronal activity, when glucose metabolism is too slow, and hypoglycemia (Sofroniew and Vinters, 2010; Brown and Ransom, 2007; Brown et al., 2001; Pellerin et al., 1998). This has been shown in both rodents and humans (Wyss et al., 2011; Smith et al., 2003; Hu and Wilson, 1997). The role of lactate availability related in both normative functioning and psychopathology requires further investigation.

### Lactate, neural health, and bipolar disorder

The role of lactate in BD is unclear, and to the best of our knowledge, six studies have compared lactate availability between adults with BD and healthy adults. Both lower (Brady et al., 2012) and higher cingulate cortex (Machado-Vieira et al., 2017; Chu et al., 2013; Xu et al., 2013; Soeiro-de-Souza et al., 2016) and frontal cortex (Dager et al., 2004) neural lactate measurements have been reported in patients with BD compared to healthy controls (HCs) (see review Dogan et al., 2018). These discrepancies may be related to the psychiatric state of the participants, regions of interest in the brain, acquisition parameters, scanner strength, and participant activity during the scan. To the best of our knowledge, all published studies on BD have examined lactate availability during rest; however, to reduce the burden of long magnetic resonance spectroscopic imaging (MRSI) scan acquisitions, participants are often allowed to watch a movie, which is a standard practice in many imaging centers (personal communication, Hetherington). We contend that watching a movie does not constitute rest and is, in fact, consistent with emotion processing. Studies on lactate availability in rodents (Hu and Wilson, 1997) and in healthy volunteers, measured using MRSI (Koush et al., 2019; Schaller et al., 2014; Kuwabara et al., 1995) and PET (Prichard et al., 1991), have suggested that lactate availability fluctuates in the associated neural regions during both cognitive and emotional task activities. Therefore, an exploration of lactate availability during both rest and task performance is essential. Our small preliminary dataset is presented to highlight the potential role of lactate flux in understanding BD.

In this study, we focused on the precuneus (Broadman area 7), the medial portion of the parietal cortex, which is implicated in BD and identified in both the default mode network (DMN) and the frontoparietal network (FPN). This indicates its crucial role in functions during both rest and tasks that require high glucose metabolism and energy efficiency (Cavanna and Trimble, 2006; Utevsky et al., 2014). We hypothesized that differences in lactate availability between task and rest (lactate flux) represent a tightly regulated system crucial for neural function and that greater flux would be observed in patients with BD than in healthy controls. We also hypothesize that, in line with the literature, during the emotion processing task, patients with BD would show elevated precuneus lactate availability. In addition, we hypothesized that patients with BD at rest would show reduced precuneus lactate availability compared to healthy participants. We further hypothesized that reduced precuneus lactate availability at rest would be negatively related to depression scores, lactate availability during the task would be positively related to mania scores, and lactate flux would be associated with both depression and mania scores.

## Materials and methods

Two sample groups were used in this study to measure precuneus lactate availability using 7-Tesla (7T) MRSI: (1) the first group comprised 26 individuals recruited for participation in a NARSAD Young Investigator Award-funded study and (2) the second group comprised 16 adolescents from the InCabs Imaging study, recruited for an internally funded pilot study. The NARSAD sample included 18 adults with well-characterized pediatric-onset BD, recruited from the Course and Outcome of Bipolar Disorders in Youth (COBY) study [mean age = 26.6 (4.53) years, of which 11 were female], and 8 healthy adults [mean age = 29.4 (8.55) years, of which 4 were female]. The internally funded pilot study included eight inpatients with pediatric-onset BD [mean age = 16.7 (1.98), six female] and eight HCs [mean age = 18.6 (3.3), five female]. All procedures were approved by the University of Pittsburgh Human Research Protection Office. Adult participants provided consent, and for child participants, parent/guardian consent along with child assent were obtained.

Participants were excluded from the 7T MRSI analysis (*n* = 16) due to acquisition difficulties, an inability to tolerate the protocol, and post-processing quality control exclusions. From the NARSAD study, nine patients with BD and six HCs were excluded. From the internally funded study, one HC was excluded. The final sample included 17 participants with BD and 8 HCs [mean age = 19.2 (3.8) years; see Table 1].

**Table 1:** Sample clinical and demographic information.

		Narsad sample		InCabs sample		Statistics	*p*-value	BD (*n* = 18)	HC (*n* = 8)	BD (*n* = 8)	HC (*n* = 8)
Age (years)		26.6 (4.53)	29.4 (8.55)	16.17 (2.32)	16.33 (1.15)	t(27) = 0.52	0.959
Gender (Female)		11	4	5	2	X2 = 0.068	0.794
	With usable MRSI data						*n* = 9	*n* = 1	*n* = 8	*n* = 7		
	Current mood state based on self report of usable MRSI data							
Euthyic		3 (33%)		0			
Depressed		3 (33%)		3 (37.5%)			
Manic		2 (22%)		0			
Mixed	1 (11%)		5 (62.5%)			
Psychotropic medication use (yes)		4	0	6	0		

BD, pediatric onset bipolar disorder; HC, healthy control. Number of participants in current mood state: depression state derived from the Center for Epidemiologic Studies Depression Scale or Mood and Feelings questionnaire as appropriate for age. Mania state derived from the Altman Self Rating Mania Scale or Brief mania rating scale as appropriate for age. Mean (standard deviation) or n (percentage) reported as appropriate.

We assessed self-reported depression-related energy using the Center for Epidemiologic Studies Depression Scale (CES-D) (Radloff, 1977) and the Mood and Feelings Questionnaire (Burleson Daviss et al., 2006). We assessed self-reported mania-related energy using the Altman Self-Rating Mania Scale (ASRM) (Altman et al., 1997) and the Brief Mania Rating Scale (Pavuluri et al., 2006), as appropriate for participant age. Higher scores on these scales indicate more severe symptoms. Based on self-report, the current mood states of the participants with BD were as follows: euthymic (*n* = 3), depressed (*n* = 6), manic (*n* = 2), and mixed (*n* = 6).

7T MRSI acquisition (Lin et al., 2021): ^1^H spectroscopy data were acquired (Pan et al., 1998; Pan et al., 2010; Pan et al., 2012; Pan et al., 1996; Pan et al., 2000; Pan et al., 2021; Hetherington et al., 2010) using a 7T Siemens Magnetom whole-body human scanner with 32 independent receiver channels and 8 independent transmit channels, utilizing a 16-channel transceiver array and a very high order shim (VHOS) system. The pTx system and transceiver coil facilitate B1 + shimming in a targeted slice of the brain, while a homogeneous B0 shim is achieved using the VHOS system. In addition to enabling a homogeneous B1 + shim in the slice of the brain, the unique design of the transceiver coil—tapered to match the head shape—creates a ring-shaped B1 + distribution for the suppression of extracerebral fat tissues, which is critical for MR spectroscopy to remain uncontaminated by large fat signals. B0 shimming was performed using a combination of Siemens first- and second-order shims and VHOS third- and fourth-order shims. On-site, per-subject B1 + and B0 shim calculations were carried out using a dedicated computer connected to the scanner network. The applied MRSI J-refocused lactate editing sequence was a single BASING sequence with an editing radiofrequency (RF) pulse (Harris et al., 2017) at 4.1 ppm, requiring homogeneous B1 + and B0 shim performance. Sequence imaging was encoded using a highly efficient rosette k-space trajectory for spatial–temporal encoding while using a minimal maximum gradient amplitude of 16 mT/m and a slew rate of 120mT/m to minimize potential eddy current artifacts. The imaging parameters were as follows: TR/TE = 1500/45 ms, bandwidth = 2,500 Hz, readout duration = 320 ms, matrix size = 16 × 16, pixel size = 13.5 × 13.5 × 9mm^3^, and total sequence time = 3:12, with two interleaves and EDIT-On/Off conditions. The sequence was repeated four times and averaged to increase the SNR. Multi-channel spectroscopy data were combined with the correct weight and phase per channel, using matched scout proton imaging. MPRAGE images (TR = 3,500 ms, TE = 3.02 ms, TI = 1,200 ms) were used for anatomical identification. Planar MRSI data (TE = 45 ms, TR = 1,500 ms, FOV = 216 × 216 mm^2^, slice thickness = 9 mm, voxel size = 13.5 × 13.5 × 9 mm^3^) were acquired in a single axial slice containing the precuneus, using a slice-selective J-refocused coherence transfer sequence.

### MRSI paradigms

#### Resting state

Participants were instructed to stay awake and look at a fixation cross during the resting state acquisition. Each block was 3 minutes 12 seconds. We averaged over 4 blocks to improve SNR.

#### Emotion processing task

The participants were instructed to respond to emotional faces (the faces represented fear, happiness, anger, and neutrality) presented on a screen by pressing a button to indicate the color of the background. Each block was 3 minutes 12 seconds. We averaged over 4 blocks to improve SNR. This block design task has been used in functional neuroimaging paradigms with both children and adults (Greenberg et al., 2017; Acuff et al., 2018; Steele et al., 2021; Oppenheimer et al., 2021; Schumer et al., 2024) and reliably activates emotion processing regions. Task performance over the entire time series contributes to the lactate measurement and may serve as an indicator of lactate flux capacity during daily activity interactions.

#### Spectral analysis

The lactate signal in two manually selected precuneus voxels was extracted from differential data, i.e., the subtraction of Lactate EDIT-Off from EDIT-On. The spectrum was fitted to LCModel (LCM) for quantification, as described in the study by Pan et al. (2000). Spectral analysis was performed using LCM with tissue classification based on MP2RAGE images. A bandwidth of 1.8–6.6 ppm (omitting the water region) was analyzed using two compound basis functions (creatine and lactate), calculated from GAMMA simulations incorporating the semi-selective refocusing profile. The SNR was calculated as the peak-to-peak amplitude divided by standard deviation of the downfield 150 spectral points from the residual spectrum; linewidth was determined using LCM. The basis spectra for the editing sequence were calculated from the freely available GAMMA C++ library for simulating MR experiments (Smith et al., 1994). Creatine was used as the reference, given the minimal variation in the cr-phosphocreatine (Cr-PCr) equilibrium, resulting in a stable concentration of Cr (Scotti-Muzzi et al., 2021). The Cramer–Rao lower bound (CRLB) values for each ratio were used to assess spectral quality.

We used a two-tailed, two-sample *t*-test (*p* < 0.05) and Bayes factor_10_ (BF_10_) statistics to assess between-group differences. We assessed the relationships between self-reported depression and mania symptoms using linear regression. All analyses were conducted using JASP (Goss-Sampson, 2019; Wagenmakers et al., 2018; Love et al., 2019; Hu et al., 2018).

## Results

**Figure 1:**
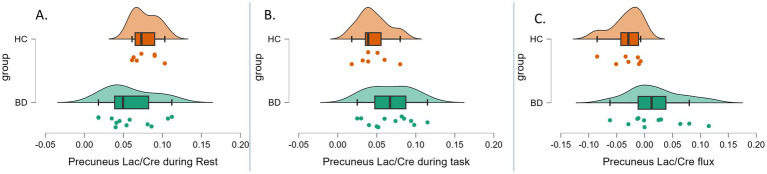
Raincloud plots of precuneus lactate availability acquired with MRSI. **(A)** During rest (duration 12 min 48 s), *t*(22) = −1.51, *p* = 0.146, Cohen’s d = 0.652, and BF10 = 0.855. **(B)** During an emotion processing task (duration 12 min 48 s), *t*(18) = 1.72, *p* = 0.102, Cohen’s d = 0.807, and BF10 = 1.092. **(C)** Lactate flux: the difference between precuneus Lac/Cre availability during the task and precuneus lactate availability during rest [*t*(17) = 2.64, *p* = 0.017, Cohen’s d = 1.26, BF10 = 3.528]. Lac/Cre, ratio of lactate to creatine; BD, bipolar disorder, data points in green, HC, healthy control, data points in orange.

In this preliminary analysis, precuneus Lac/Cre availability flux (task–rest) differed significantly between the patients with BD [mean Lac/Cre = 0.014 (0.041)] and healthy controls [mean Lac/Cre = −0.03 (0.028)], *t*(17) = 2.64, *p* = 0.017, Cohen’s d = 1.26, and BF₁₀ = 3.528 (Figure 1C). Precuneus Lac/Cre availability during the emotion processing task did not differ between patients with BD [mean = 0.07 (0.03)] and healthy controls [mean = 0.05 (0.02), *t*(18) = 1.72, *p* = 0.102, Cohen’s d = 0.807, BF_10_ = 1.092]. Similarly, no significant difference was observed during rest between patients with BD [mean = 0.06 (0.029)] and healthy controls [mean = 0.07 (0.022), *t*(22) = −1.51, *p* = 0.146, Cohen’s d = 0.652, BF_10_ = 0.855] (Figures 1A,B).

### The relationship between precuneus lactate availability and current depression symptoms

**Figure 2:**
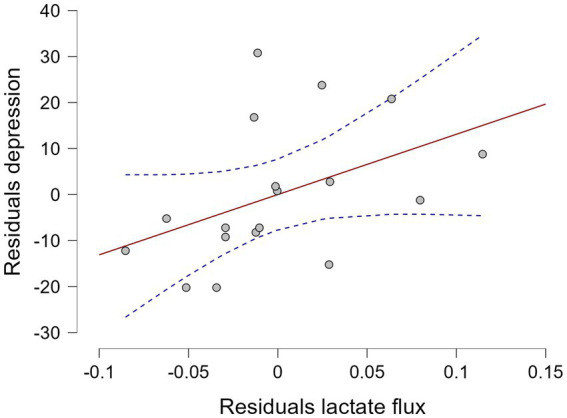
Relationship between precuneus lactate flux and self-reported depression scores on the day of the MRSI scan [*F*_(1, 16)_ = 4.27, *p* = 0.055, *r*^2^ = 0.211, BF10 = 1.617].

Lactate availability flux (task–rest) positively predicted self-reported depression scores at the trend level: *F*_(1, 16)_ = 4.27, *p* = 0.055, *r*^2^ = 0.211, BF_10_ = 1.617 (Figure 2). However, lactate availability during the task [*F*_(1, 17)_ = 1.192, *p* = 0.290, *r*^2^ = 0.066, BF_10_ = 0.609] and during rest [*F*_(1, 21)_ = 3.717, *p* = 0.067, *r*^2^ = 0.150, BF_10_ = 1.00] did not predict depression scores.

### The relationship between precuneus lactate availability and current mania symptoms

Self-reported mania scores were not predicted by lactate availability flux [*F*_(1, 17)_ = 0.170, *p* = 0.685, *r*^2^ = 0.010, BF_10_ = 0.429]. In addition, lactate availability during the task [*F*_(1, 18)_ = 0.259, *p* = 0.617, *r*^2^ = 0.014, BF_10_ = 0.461] and during rest [*F*_(1, 22)_ = 1.695, *p* = 0.206, *r*^2^ = 0.072, BF_10_ = 0.685] did not predict mania symptom scores.

## Discussion

To the best of our knowledge, this is the first study to examine neural lactate availability during both rest and task activity, as well as lactate availability changes from rest to task activity (lactate flux), in well-characterized young adults with pediatric-onset BD. These preliminary results demonstrate abnormalities in precuneus lactate availability, particularly in the availability and use of lactate between task activity and rest (flux), compared to healthy controls. In both patients with BD and healthy participants, self-reported depression scores were positively associated with lactate flux, but their relationships with self-reported mania were weak. The positive relationship between lactate flux and depression suggests that lower lactate availability at rest, followed by a substantial increase in lactate availability during the task, contribute to this association. Future studies can explore these relationships in relation to sedentary behaviors in individuals with BD.

Specifically, we found that the difference in lactate availability was greater in participants with BD than in the healthy controls. Lactate flux in the participants with BD ranged from −0.062 to 0.080, while in the healthy participants, it ranged from −0.085 to −0.007. All healthy participants had greater lactate availability during rest than during the task in the precuneus, the task-associated region (Bruner et al., 2017; Cavanna and Trimble, 2006). Normative reduced lactate availability during tasks is consistent with studies involving rodents (Wyss et al., 2011; Holloway et al., 2007; Hu and Wilson, 1997) and humans (Sofroniew and Vinters, 2010; Smith et al., 2003). These findings may directly align with the understanding of lactate as the end product of glycolysis (Schurr, 2006; Schurr, 2023; Schurr et al., 1999; Schurr and Payne, 2007; Schurr et al., 1997a; Schurr et al., 1997b), the understanding of lactate as the preferred energy source for neurons (Smith et al., 2003; Bouzier-Sore et al., 2003; Wyss et al., 2011), and the idea that lactate is directly associated with adaptive cognitive functioning (Schurr et al., 1997a; Schurr et al., 2001). Our findings suggest that, in healthy individuals, lactate is produced and used on demand to fuel neurons, which is consistent with other studies (Hu and Wilson, 1997; Mächler et al., 2016). The abnormal increases in lactate during the task observed in participants with BD in this study may parallel experimental findings in rodents, where elevated *in vivo* lactate levels have been shown to alter neuronal pH, potentially disrupt action potential conductance, and potentially delay response times (Wyss et al., 2011). The abnormal increases in lactate during typical human task-related behavior observed in this study suggest dysfunction in neural lactate metabolism flux. This may be associated with dysfunction between astrocytes and neurons in monocarboxylate transporter (MCT) functioning, lactate dehydrogenase (LDH) availability, or glutamate signaling.

We examined lactate availability in the precuneus due to its functional involvement in both the DMN and the FPN and its high metabolism (Cavanna and Trimble, 2006; Utevsky et al., 2014). The precuneus is a key hub in both the DMN—a task-negative network associated with introspection—and the FPN—a task-positive network associated with visuospatial memory/processing, attention, and flexible goal-directed behaviors. Specifically, how local metabolism drives regional functional connectivity (FC) within and between networks is not completely understood (Voigt et al., 2023; Jamadar et al., 2021); however, a recent study demonstrated that high FPN metabolic activity is normatively maintained during rest, possibly to allow for efficient switching between task engagement and rest (Jamadar et al., 2021). Indeed, precuneus hypometabolism observed in patients with mild cognitive impairment (Mattioli et al., 2021) may be associated with deficits in efficient network switching. The abnormal lactate flux associated with depression scores observed in this study in a key DMN and FPN hub may reflect a deficit in precuneus lactate availability during rest—potentially impairing network switching—and may be related to emotion processing abnormalities observed in BD, particularly during depressive episodes. Furthermore, aberrant between-network FC during rest has been implicated in BD, with abnormal precuneus FC—particularly with the salience network involved in emotion processing (Singh et al., 2015; Zhao et al., 2024; Lopez-Larson et al., 2017; Zhang et al., 2025)—supporting findings of disrupted DMN integrity and between-network FC in BD (Uddin et al., 2009). Larger studies are needed to confirm these results.

Our findings did not support our hypotheses concerning the specificity of lactate availability during rest and task, which may be due to our small sample size and should be examined in larger samples containing patients with well-characterized BD and healthy controls. Our findings also did not support our hypothesis that precuneus lactate availability during the task would be positively related to mania scores. While this is unexpected, lactate availability in other neural regions may be implicated in manic behaviors.

**Figure 3:**
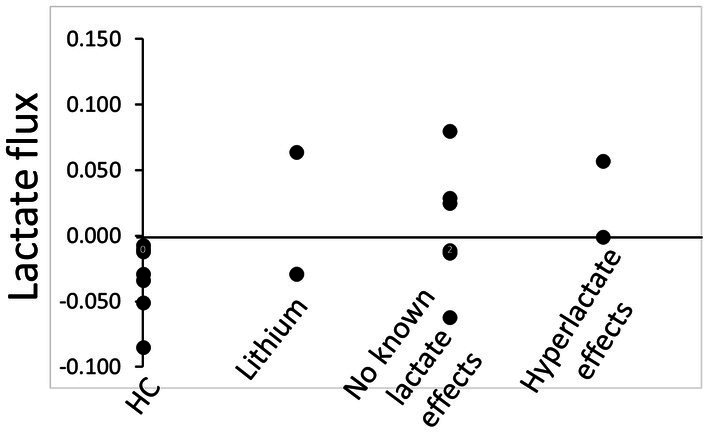
Psychotropic medication use and precuneus lactate flux on the day of the MRSI scan. HC, healthy control. Only BD participants reported taking psychotropic medications.

There are limitations to this study. The sample size was small, but we used Bayesian models to better interpret our results. The effect sizes suggest that testing lactate availability and lactate flux from task to rest in patients with BD warrants further investigation. Although our participants were medicated, those medications did not systematically influence the lactate flux measure in this study (Figure 3).

Our preliminary findings highlight the important, potentially mechanistic role of precuneus lactate dysfunction during rest and task in patients with BD. If further supported by other studies, our results could be used in developing novel treatment options for BD that target lactate availability and flux in key neural regions. These novel treatments may target lactate availability directly, such as those being tested to treat traumatic brain injury (Holloway et al., 2007; Glenn et al., 2015), or may address the underlying mechanisms of lactate metabolism or astrocyte dysfunction.

## Data Availability

The raw data supporting the conclusions of this article will be made available by the authors, without undue reservation.
